# Biofilm Characterization by AFM and SEM and Growth Kinetics of *Geobacter sulfurreducens* in Regional Cheese Whey

**DOI:** 10.3390/microorganisms14071414

**Published:** 2026-06-27

**Authors:** Juana Elizabeth Alba-Cuevas, Virginia Villa-Cruz, Héctor Pérez Ladrón de Guevara, Lily X. Zelaya-Molina, Haiku Daniel Gómez-Velázquez

**Affiliations:** 1 Centro Universitario de los Lagos, Universidad de Guadalajara, Lagos de Moreno 47460, Mexico; elizabeth.alba@academicos.udg.mx (J.E.A.-C.);; 2 Centro Nacional de Recursos Genéticos-INIFAP, Tepatitlán de Morelos 47600, Mexico

**Keywords:** *Geobacter sulfurreducens*, cheese whey, Atomic Force Microscopy (AFM), electroactive biofilms, waste valorization

## Abstract

*Geobacter sulfurreducens* is a model bacterium widely used in microbial fuel cell (MFC) research due to its efficient extracellular electron transfer. However, the high cost of synthetic media limits the scalability of these systems, making agro-industrial byproducts like cheese whey a sustainable alternative. This study evaluated cheese whey as a growth medium for *G. sulfurreducens* and its influence on biofilm development on graphite bars electrodes. Bacterial growth kinetics and biofilm architecture were characterized using Atomic Force Microscopy (AFM) as the primary quantitative tool, supplemented by Scanning Electron Microscopy (SEM). Growth curves revealed a diauxic-like transition within the first 48 h, with high cell viability (94%). AFM analysis demonstrated a non-linear topographical evolution: an initial attachment phase was followed by a peak in structural heterogeneity at 14 days (*S*_q_ = 683.08 nm), eventually reaching a mature, confluent state at 21 days with a maximum thickness of ~8 μm. Energy-Dispersive Spectroscopy (EDS) confirmed an organic and mineral matrix consistent with bacterial biomass and whey components. These results demonstrate that cheese whey effectively supports the growth of *G. sulfurreducens* and the formation of structurally complex biofilms, highlighting its potential as a low-cost substrate for microbial cultivation and dairy waste valorization.

## 1. Introduction

The global production of cheese whey, a major agro-industrial by-product, poses a significant environmental challenge due to its high biochemical and chemical oxygen demand (BOD: 40,000–60,000 mg/L; COD: 50,000–80,000 mg/L) [1,2,3]. In Mexico alone, cheese production in 2024 is estimated at 474,000 metric tons, which corresponds to the generation of approximately 4.26 million metric tons of whey [4]. When discharged untreated, this residue leads to severe ecological imbalances and oxygen depletion in aquatic ecosystems [1,2,3]. However, due to its nutritional richness—including lactose, proteins, minerals, and vitamins—whey has been used as a cost-effective culture medium in bacterial fermentation processes and other biotechnological applications [3,4,5,6,7,8,9,10].

In the field of renewable energy, microbial fuel cells (MFCs) have emerged as a promising technology for waste valorization [11]. *Geobacter sulfurreducens* is widely used as a model organism in MFC research due to its capacity for extracellular electron transfer and biofilm formation on conductive surfaces [11]. These biofilms can reach thicknesses of up to 130 µm and establish crucial electron transfer pathways across the anode surface [12,13,14]. The extracellular electron transfer (EET) metabolism of *G. sulfurreducens* involves a coordinated network of conductive pili (nanowires), outer-membrane cytochromes, and extracellular polymeric substances (EPS), which collectively facilitate electron transport within the biofilm matrix and between the biofilm and conductive surfaces [10,15,16,17,18].

Traditionally, bacterial morphological analysis has relied on optical microscopy [3]. However, the limited resolution of these methods restricts the detailed examination of surface structures. Consequently, high-resolution techniques such as scanning electron microscopy (SEM) and atomic force microscopy (AFM) have become essential for ultrastructural analysis [4]. AFM, in particular, offers a unique advantage by providing quantitative topographical data, such as surface roughness and height distribution, which are critical for understanding how the culture medium influences microbial phenotypic plasticity and adaptive strategies [19,20,21,22].

Despite the recognized potential of whey as a sustainable substrate and the importance of biofilm architecture in the development of *G. sulfurreducens* communities on conductive materials, the specific impact of whey-based cultivation on the ultrastructural characteristics and surface topography of *G. sulfurreducens* biofilms remains largely unexplored. While most previous studies have relied on defined synthetic media or focused primarily on the short-term electrochemical outputs of waste-fed systems, the physical dynamics of the biofilm itself are often overlooked. In particular, limited information is available regarding how a complex agro-industrial byproduct like cheese whey influences long-term biofilm maturation and surface topography. We hypothesized that the nutritional composition of cheese whey would support the growth of *G. sulfurreducens* and promote measurable structural changes during biofilm development on graphite surfaces. Therefore, the present study aimed to analyze the surface morphology and temporal dynamics of *G. sulfurreducens* biofilms cultivated on cheese whey using graphite bar electrodes. High-resolution SEM and AFM were used to quantitatively characterize the morphological adaptation and structural maturation of the biofilm over a 21-day period. The results document the structural changes observed during the development of *G. sulfurreducens* biofilms in cheese whey and provide a fundamental morphological baseline for future studies on the use of alternative substrates for microbial growth and biofilm formation.

## 2. Materials and Methods

### 2.1. Whey Source, Sampling, and Composite Substrate Preparation

Whey samples were obtained from five distinct local dairy producers located in the Altos Norte region of Jalisco, Mexico: Producer A, Producer B, Producer C, Producer D, and Producer E. A total of 90 independent samples were gathered from different production batches immediately after the cheese curdling process. All samples were collected in sterile containers and transported to the laboratory under refrigeration (4 °C within 2 h of collection for immediate screening).

To evaluate the regional agro-industrial waste stream while ensuring maximum experimental reproducibility during *Geobacter sulfurreducens* colonization assays, two parallel strategies were implemented: (i) individual samples from each manufacturer were preserved for independent compositional screening, and (ii) a standardized composite lot (pooled master batch) was prepared by mixing equal volumes of fresh whey from all five local producers. This pooling approach was specifically designed to mitigate the inherent compositional variability of cheese whey—driven by seasonal factors, cattle diet, and distinct artisanal processing methods—thereby establishing a representative and reproducible nutritional baseline. This composite matrix, representing the average profile of regional sweet whey, was chemically characterized to track key macronutrients essential for microbial metabolism, revealing substantial natural concentrations of phosphorus (P) and potassium (K), which eliminate the need for synthetic mineral supplementation. Subsequently, the composite matrix was completely sterilized by autoclaving at 121 °C for 15 min [23,24,25], to eliminate native microbiota and ensure monoxenic conditions. Following sterilization, the medium was centrifuged at 500 rpm (approximately 23× *g*) for 10 min using a Benchmark LC-8 centrifuge (Benchmark Scientific, Sayreville, NJ, USA); the supernatant was collected to remove suspended fats and insoluble precipitates that could interfere with biofilm attachment. The pH of the sterilized medium was adjusted to 6.0 according to [26]. The sterilized bulk substrate was subsequently stored under refrigeration (4 °C) to serve as the uniform, sterile culture medium stock for all subsequent biological replicates and biofilm attachment experiments.

### 2.2. Physicochemical Characterization and Statistical Analysis

Prior to the biological essays, the macromolecular composition of the individual whey samples and the master batch was determined in triplicate. Lactose, Total protein content, Total fat content was evaluated using a Milkoscan™ Mars FTIR spectrometer (FOSS, Hillerød, Denmark). Detailed chemical composition was determined via FTIR spectroscopy using a MilkoScan FT1 (FOSS, Denmark) [27,28,29]. Global prediction models for cheese whey, compliant with ISO 9622 and IDF 141 standards, were employed. To ensure measurement consistency, the instrument was operated following the manufacturer’s cleaning and automatic standardization (zero-setting) protocols.

To determine whether the distinct whey sources could be considered compositionally equivalent, a comprehensive screening analysis was performed. Data normality was first verified using the Shapiro–Wilk test. Subsequently, a one-way Analysis of Variance (ANOVA) followed by Tukey’s post hoc multiple comparison tests (α = 0.05) were implemented to evaluate the compositional variability among the different dairy producers. All statistical analyses were conducted using IBM SPSS statistical software, version 29.

### 2.3. Bacterial Strain and Culture Conditions

The strain ATCC 51573 of *Geobacter sulfurreducens*, obtained from the American Type Culture Collection (ATCC, Manassas, VA, USA), was used in this study. This bacterium is classified as a biosafety level 1 (BSL-1) organism and is not known to cause disease in healthy adults. The lyophilized strain was reconstituted and propagated in ATCC 1957 medium (*Geobacter* medium). To establish and maintain the strict anoxic conditions required for this obligate anaerobe without the addition of artificial chemical reducing agents, all microbiological manipulations, vial reactivations, and subsequent inoculations into the standardized pooled whey substrate (Section 2.1) were conducted inside an anoxic chamber. The internal atmosphere of the chamber was completely displaced and continuously sustained with CO_2_ gas. The anoxic environment and gas concentration within the workstation were strictly verified and maintained using a portable gas monitoring analyzer to ensure a constant, oxygen-free headspace during all experimental setups.

Following initial propagation, a cell suspension was harvested and standardized to a concentration of approximately 1.0 × 10^8^ cells/mL by adjusting the optical density to the McFarland 0.5 standard (≈1.5 × 10^8^ cells/mL equivalent). Serial dilutions were subsequently performed inside the anoxic chamber using sterile, deoxygenated saline solution (0.85% NaCl) to achieve a final target inoculum concentration of approximately 10^4^ cells/mL. To ensure experimental reproducibility and the physiological robustness of the biological catalyst, only bacterial suspensions demonstrating a post-activation cell viability higher than 94% were selected for subsequent assays. This viability threshold was strictly verified using an automated Corning cell counter (model 6749, Tewksbury, MA, USA) via the Trypan blue exclusion staining method. This standardized inoculum was immediately transferred into the treated composite whey medium.

### 2.4. Anaerobic Bioreactor Configuration for Biofilm Cultivation

The cheese whey-based medium was introduced into a dual-chamber glass vessel originally designed as a microbial fuel cell (MFC) (model MFC100.25.3, Adams & Chittenden Scientific Glass, Berkeley, CA, USA). However, for the purposes of this study, the system was operated strictly as a specialized anaerobic bioreactor to investigate biofilm hosting and development The device was equipped with graphite rod electrodes (15 cm^2^ total area, with a submerged working area of 9 cm^2^) which served exclusively as a conductive, solid physical support for *Geobacter sulfurreducens* colonization. Throughout the evaluation period, the system was maintained predominantly under unpolarized, resting electrical conditions, allowing the biofilm to establish and mature natively on the substrate during the course of the experiment.

### 2.5. Growth Kinetics

Growth of *G. sulfurreducens* in whey medium was monitored via optical density (OD_600_) in transparent 96-well polystyrene plates. An initial inoculum of 1 × 10^4^ cells/m, previously cultured for 10 days in whey medium, was used. Each well contained 293 μL of culture broth and 3 μL of bacterial inoculum. Assays were performed in triplicate. Growth was recorded automatically every 15 min for 10 days using a Multiskan Go microplate spectrophotometer (model 1510-01413C, Thermo Scientific, Waltham, MA, USA). Plates were incubated at 36 °C under static conditions to favor initial cell adhesion and structured biofilm development [30], allowing for the establishment of local nutrient, pH, and redox gradients [31,32]. These conditions promote mature and thicker biofilms, enhancing EPS production and physiological differentiation [32,33]. A control consisting of sterile broth with 3 μL of sterile water was included.

### 2.6. Biofilm Formation and AFM Analysis

Sterile graphite bars were placed in a double-chamber MFC with a contact area of approximately 8 cm^2^ with the inoculated whey. After incubation, the bars were removed and air-dried at room temperature for 7 days inside an anaerobic chamber. AFM analysis was performed using a NanoSurf EasyScan 2 atomic force microscope (Nanosurf AG, Liestal, Switzerland) mounted on an anti-vibration workstation. Measurements were performed with a 70 μm scanning head in contact mode. Images were initially obtained at a 70 × 70 μm^2^ scanning area before zooming into specific regions of interest [34]. Surface roughness (S_q_) was determined using the Nanosurf EasyScan 2 software, and surface skewness (S_sk_) was calculated using Gwyddion software (v2.60) over the entire 68 × 68 μm^2^ area to ensure statistical representative results.

### 2.7. Environmental Scanning Electron Microscopy (ESEM) and EDS Analysis

Graphite bars were removed from the liquid medium, washed three times with PBS to remove non-adherent cells, and air-dried for 7 days in an anaerobic chamber. Samples were sputter-coated with a thin gold layer (~20 nm) using a 99.9% purity gold target. Morphological characterization was performed using an environmental scanning electron microscope (Philips/FEI XL30 ESEM, Eindhoven, The Netherlands) at an acceleration voltage of 1.0 kV for imaging. Multi-elemental analysis (EDS) was conducted using an XFlash^®^ 6/10 silicon drift detector (Bruker Nano GmbH, Berlin, Germany) at 15 kV. Data were processed using Fiji/ImageJ software version 1.54g [35].

## 3. Results

### 3.1. Characterization of Whey as a Culture Medium

The macromolecular compositional profile of the initial cheese whey screening (n = 90) and the standardized composite lot used for the experimental trials are summarized in Table 1 and Table 2, respectively. The preliminary screening revealed that lactose was the predominant component with a mean concentration of 3.90%, followed by total protein (1.29%) and fat (1.05%), resulting in an overall total solids content of 6.54%.

To evaluate the compositional variability among the five local dairy suppliers, a one-way ANOVA was conducted (Table 1). The statistical analysis demonstrated highly significant differences (*p* < 0.001) among producers across all evaluated variables. The highest variation between producers was observed in the fat content (F4,85 = 97.379, *p* < 0.001), followed by lactose (F4,85 = 15.473, *p* < 0.001) and total solids (F4,85 = 15.361, *p* < 0.001). Protein content also exhibited significant differences among suppliers (F4,85 = 11.639, *p* < 0.001).

The standardized composite lot prepared to perform the subsequent kinetic and biofilm assays showed a stabilized physicochemical profile (Table 2). This matrix presented a lactose content of 4.422 ± 0.009%, total protein of 1.062 ± 0.005%, total fat of 0.9 ± 0.008%, and total solids of 6.54 ± 0.061%. The reference literature values for the regional context regarding Chemical Oxygen Demand (COD) and Biochemical Oxygen Demand (BOD) ranged between 50,000–80,000 mg/L and 37,000–60,000 mg/L, respectively.

### 3.2. Growth Kinetic Parameters

**Figure 1:**
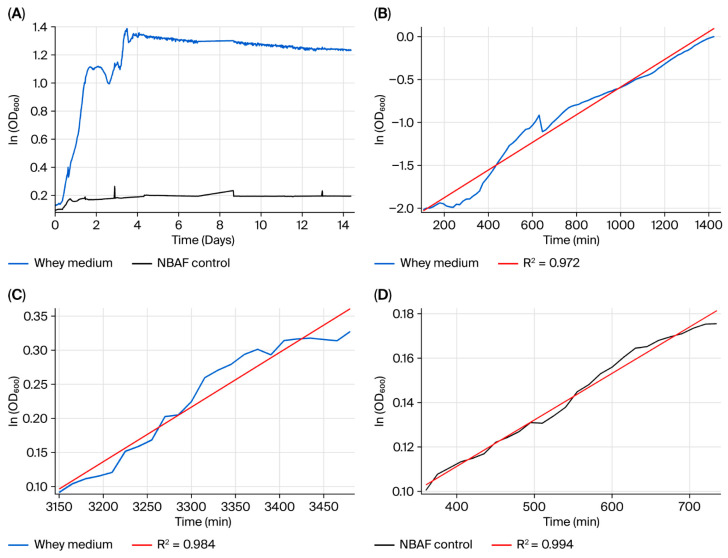
Growth kinetics of *G. sulfurreducens* ATCC 51573 in whey-based medium. (**A**) Growth curves monitored via optical density (OD_600_) comparing whey medium versus NBAF control; (**B**) Linear regression of the first exponential phase in whey (R^2^ = 0.972); (**C**) Linear regression of the second exponential phase in whey (R^2^ = 0.984); (**D**) Linear regression of the NBAF control (R^2^ = 0.994). Error bars represent the standard deviation (*SD*, n = 3) for the triplicate assays.

The growth dynamics of *G. sulfurreducens* ATCC 51573 in the whey-based medium were evaluated by monitoring optical density (OD_600_) over time (Figure 1A). The growth curve exhibited an initial lag phase followed by an exponential phase lasting approximately 24 h. Subsequently, a plateau and a subsequent diauxic-like exponential shift were observed at 48 h before the culture entered a stable stationary state.

To determine the specific growth rate (μ), the data from each exponential phase were linearized by plotting the natural logarithm (ln) of OD600 against time for both the whey medium and the NBAF control (Figure 1B–D). The specific growth rate (μ, h^−1^) was derived from the slopes of the resulting linear regressions, which yielded correlation coefficients of R^2^ = 0.972 for Phase I and R^2^ = 0.984 for Phase II. Based on these values, additional kinetic parameters, including doubling time (g) and the number of generations (n), were calculated for each phase and are summarized in Table 3. All values are expressed as the mean ± standard deviation (SD) of triplicate experiments (n = 3).

### 3.3. Morphological and Topographical Analysis of Biofilm (AFM)

**Figure 2:**
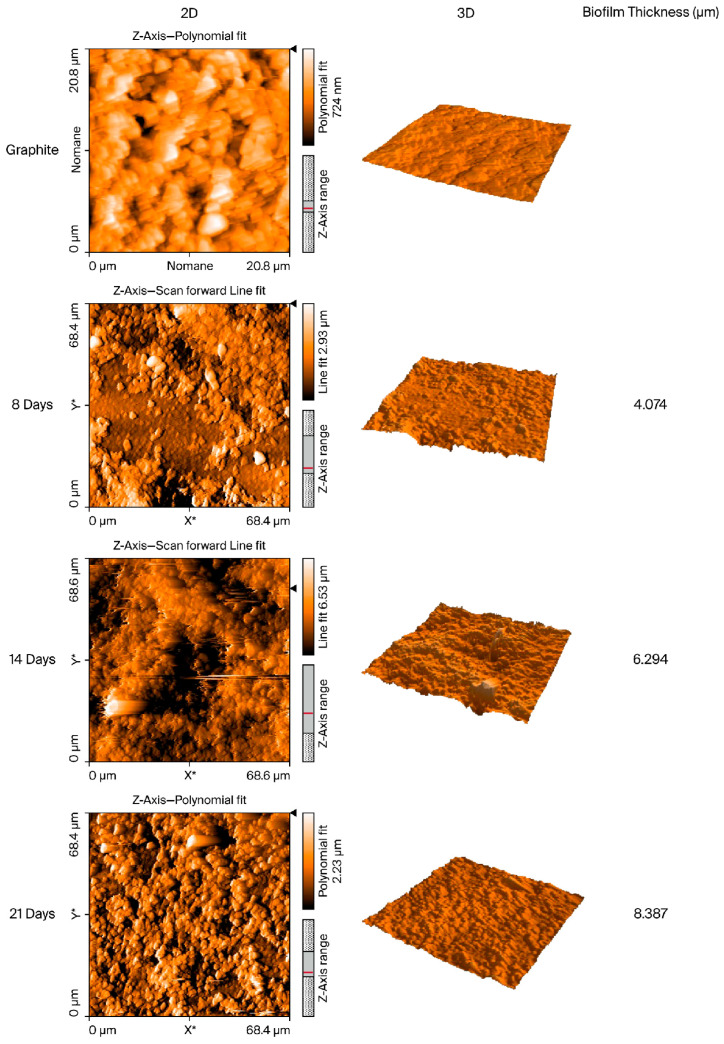
Spatiotemporal evolution of *G. sulfurreducens* biofilm morphology and topography on graphite bar electrodes. The panel is organized into four rows representing the colonization stages: control (bare graphite bar), 8, 14, and 21 days of incubation in whey-based medium. Columns correspond to: (1) 2D AFM height maps; (2) 3D surface topographical reconstructions; and (3) representative cross-sectional thickness profiles. All images were acquired at a 70 × 70 μm^2^ scan size using contact mode. The thickness profiles (Z-axis) illustrate the vertical growth and stratification of the biological matrix over time.

The developmental stages of the *G. sulfurreducens* biofilm were analyzed using AFM at 8, 14, and 21 days of incubation, using bare graphite bar as a control. The results are presented in Figure 2, organized into 2D topography (first column), 3D surface reconstruction (second column), and cross-sectional thickness profiles (third column).

The first row displays the control graphite electrode (without *G. sulfurreducens*), showing a predominantly flat and uniform topography. The 3D reconstruction and the thickness profile for the control confirm the absence of biological structures, providing a reference for the subsequent colonization stages.

At 8 days (second row), the 2D and 3D images revealed the initial attachment of bacterial cells, forming isolated primary communities. At this stage, colonization was not yet confluent, leaving significant areas of the graphite electrode exposed. The corresponding thickness profile in the third column confirmed an initial biofilm height of approximately 4.074 µm.

By 14 days (third row), a progressive expansion of the biofilm was observed. The 2D and 3D micrographs showed that the cells had begun to merge into larger aggregates, covering most of the electrode surface. The 3D reconstruction highlights an increase in surface complexity and the appearance of “shadowed” regions, which correlate with the vertical growth of the biological matrix. The corresponding thickness profile in the third column confirmed an initial biofilm height of approximately 6.294 µm.

At the final stage of 21 days (fourth row), the images illustrate a fully developed and stratified biofilm. The 2D and 3D topographies show complete coverage of the graphite rod, characterized by multiple overlapping layers of bacteria. This visual evidence of stratification is quantitatively supported by the thickness profiles (third column), which show a significant increase in the biofilm depth, reaching values of up to 8.387 µm.

#### Quantitative Topographical Analysis (S_q_ and S_sk_)

Quantitative topographical analysis (Table 4) revealed a distinct evolution of the biofilm’s surface properties. The surface roughness (S_q_) initially increased to 300.74 ± 43.71 nm in 8 days. The highest S_q_ was observed at 14 days (683.08 ± 397.04 nm), indicating the stage of maximum structural heterogeneity. By day 21, the Sq decreased to 419.73 ± 120.46 nm, confirming that the biofilm reached a mature, confluent state. The surface skewness (S_sk_) remained positive throughout the entire period, reaching a maximum of 2.47 ± 0.71 at 14 days, before settling at 1.02 ± 1.28 at 21 days.

### 3.4. Morphological and Elemental Microanalysis (SEM-EDS)

**Figure 3:**
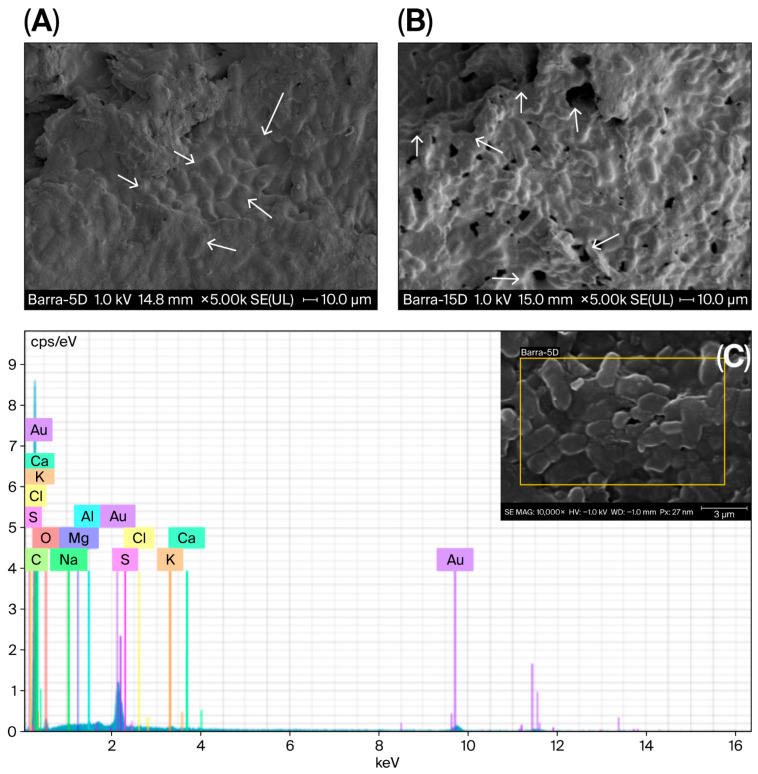
Scanning electron microscopy (SEM) and EDS microanalysis of the *G. sulfurreducens* biofilm on a graphite bar electrode. Left panel: morphological evolution captured at 1.0 kV (Scale bar: 10 µm) showing (**A**) initial colonization at 5 days, where white arrows indicate *G. sulfurreducens* cells embedded within the whey-derived organic matrix; and (**B**) a mature, stratified biofilm at 15 days, with white arrows highlighting darker regions corresponding to increased topographical depth and structural complexity. Right panel (**C**): EDS spectrum (0–16 keV) displaying the elemental composition of the biological matrix and dairy-derived precipitates.

The results of biofilm observation using SEM show that, during the first few days, the cells are scattered both in the medium and on the surface of the electrode (Figure 3A). On the final day of observation (Figure 3B), small microbial communities covering a larger surface area of the electrode can be seen, corresponding to a mature biofilm typical of *Geobacter*. An important aspect to note is that the images show that *G. sulfurreducens* bacteria are embedded in the whey, which forms a kind of surface “layer” over the electrode and microorganisms, corresponding to a stratification of the biofilm that is clearly visible in the microscopic images.

Energy Dispersive X-ray Spectroscopy (EDS) was conducted alongside SEM to determine the elemental composition and relative distribution within a specific area of the adhered biomass, as shown in Figure 3C. This analysis was crucial to confirm that the structures colonizing the surface are indeed of biological origin (the *G. sulfurreducens* biofilm) rather than inorganic precipitates or artifacts derived from the complex whey matrix. While elements typical of biological systems were detected, trace elements often associated with *Geobacter* metabolism (such as iron) may appear at very low concentrations or remain undetected due to the inherent detection limits of the EDS technique for biological matrices. Ultimately, although this elemental profile strongly suggests the presence of cellular components, EDS provides strictly elemental and semi-quantitative data; therefore, further biochemical analyses are required to conclusively identify specific extracellular polymeric substances (EPS) or complex biomolecules.

The EDS spectrum is consistent with a bacterial biofilm on graphite, as indicated by the strong signal from carbon (65.49% normalized weight percentage) and oxygen (3.20%) reflecting the organic matrix (biomass). Elements such as Na (0.09%), K (0.55%), Ca (0.06%), Mg (0.03%), Cl (0.09%), and S (0.13%) suggesting the presence of components of the culture medium (whey) and intracellular or extracellular biological functions.

## 4. Discussion

The highly significant compositional variability observed among local dairy suppliers (Table 1) underscores the heterogeneous nature of raw cheese whey as an agro-industrial byproduct. This phenomenon is strongly tied to differences in seasonal cattle feeding, regional milk processing conditions, and the specific cheese-making technologies employed by local producers [37]. In complex microbiological studies, such raw material fluctuations introduce experimental noise that can drastically impair the reproducibility of cellular adhesion and subsequent biofilm architecture [38]. By implementing a stabilization protocol, the resulting composite lot (Table 2) achieved remarkable chemical consistency, as evidenced by the narrow standard deviations (<0.07%) across all macronutrients. In scalable biotechnological operations, accounting for this inherent variability is mandatory, requiring either continuous composition monitoring or industrial-scale pre-treatments, such as centralized pooling, to guarantee process standardization and predictable organic breakdown in continuous bioreactors.

This rigorous chemical stabilization of the matrix was fundamental to unraveling the precise metabolic kinetics of the micro-organism without confounding variables. While the massive organic load and values for COD and BOD (Table 2), originally reported by [36], establish a highly osmotic and demanding environment, the defined macromolecular composition of the sterile composite lot allowed for the observation of distinct metabolic phases. Specifically, the control over the initial concentrations of non-sugar components provided the baseline to understand how *G. sulfurreducens* explores alternative carbon sources natively present in the byproduct, leading to specialized growth kinetics. Furthermore, these metabolic transitions are physiologically sustained by the mineral background naturally present in sweet cheese whey. Although not explicitly quantified in our initial characterization, dairy whey is widely documented as an abundant reservoir of essential elements, particularly phosphorus (P) and potassium (K) [39,40]. From a microbial biochemistry perspective, phosphorus is indispensable for the energetic machinery of *G. sulfurreducens*, serving as a fundamental building block for nucleic acids and driving adenosine triphosphate (ATP) synthesis via respiratory phosphorylation during alternative substrate exploration [41]. Concurrently, potassium acts as the primary intracellular osmotic regulator and an essential enzymatic cofactor, which is cellularly critical for maintaining membrane potential during osmotic stress and initial cell attachment on conductive substrates [17,42]. Consequently, the natural presence of these elements in the regional whey matrix likely functioned as an intrinsic nutritional buffer, fulfilling the micro-organism’s core physiological requirements and allowing it to adapt to this demanding environment without the need for synthetic mineral supplementation.

The diauxic growth profile of *G. sulfurreducens* ATCC 51573 in whey (Figure 2) reveals its adaptability to complex effluents. This biphasic behavior is attributable to the sequential consumption of organic acids generated naturally during the cheese curdling process. Biochemically, endogenous lactic acid bacteria ferment the abundant lactose fraction primarily into lactic acid (lactate), while heterofermentative pathways and the simultaneous degradation of the matrix produce secondary metabolites, predominantly acetate [36,38]. During Phase I, the bacteria prioritize the consumption of acetate, their preferred and thermodynamically most favorable electron donor, achieving an optimal specific growth rate (μ). This preference is consistent with the genome-scale metabolic constraints described by Mahadevan et al. and the foundational physiological frameworks established by Lovley et al., which demonstrate that acetate pathways maximize ATP yield in this strain [38]. Once free acetate is depleted, the culture undergoes a metabolic reconfiguration typical of a diauxic transition, synthesizing the enzymatic machinery necessary to shift its metabolism toward alternative substrates [43]. Subsequently, Phase II reflects the consumption of the highly abundant secondary substrate, lactate.

The slower kinetics associated with lactate oxidation explain the increase in doubling time (g) reported in Table 3, a characteristic behavior of *Geobacter* when adapting to complex wastewater [44,45]. Although *G. sulfurreducens* lacks the enzymatic pathways to degrade the lactose fraction directly, it proliferates efficiently by utilizing this organic acid load from regional whey. These robust kinetics validate the feasibility of using pure cultures of this strain in systems fed with actual dairy effluents, eliminating the need to supplement with expensive synthetic media or highly refined, single-variable standard substrates like commercial sodium acetate. While pure synthetic substrates offer optimized, rapid growth kinetics due to their easily assimilable nature, the multi-nutritional organic matrix of raw cheese whey inherently serves as a self-sustaining nutrient reservoir and bypasses the need for costly external nutritional supplementation [46,47].

Under these sterile yet metabolically dynamic conditions, the development of a robust and structured biofilm, subsequently characterized via AFM and SEM, acts as a crucial biological response directly linked to the strain’s capacity for extracellular electron transfer (EET). In *G. sulfurreducens*, the spatial organization of a dense biofilm on solid supports is fundamental to establishing the interconnected cytochrome networks required for long-range electron transport [16,48]. Furthermore, the extracellular polymeric substance (EPS) matrix not only facilitates substrate colonization during both growth phases but also serves as a protective shield against the high osmotic pressure exerted by the remaining unhydrolyzed lactose (4.422 ± 0.009%) and total solids (6.54 ± 0.061%) documented in the composite lot [49]. Consequently, the biofilm architecture ensures cellular survival and efficient substrate processing within this dense industrial effluent.

The formation of electroactive biofilms of *Geobacter sulfurreducens* on graphite bar electrodes is a complex process involving successive stages of adhesion, growth, and structural maturation. Early AFM measurements performed at day 8 showed cells adhering to the graphite substrate, characterized by a localized spatial density and limited extracellular matrix formation, a behavior that closely mirrors initial colonization trends on carbonaceous supports reported previously [50,51]. Over time, a progressive transition toward clustered cellular architectures forming distinct “microcolonies” was observed at day 14. This structural accumulation caused a sharp increase in surface roughness which, as suggested by [16], marks the active phase of extracellular polymeric substance (EPS) production, an essential requirement for cell-to-cell cohesion and stable anchoring to the conductive substrate. In the latest chronological measurements obtained in this study (day 21), the biofilm exhibited almost complete coverage of the support, an irregular topography, and a denser, consolidated structure. This aligns with findings by [52,53,54], who demonstrated that the extracellular matrix functions as a structural filler between embedded cells in the mature biofilm, contributing to structural consolidation.

Beyond qualitative visualization, a rigorous quantitative analysis of AFM-derived, three dimensional (3D), topographical parameters (Table 4) was deployed to provide a deeper, nanometric insight into the structural evolution of the biofilm, successfully bypassing the optical resolution limits of conventional macroscopic fluorescence techniques. Previous studies have demonstrated that biofilm maturation is accompanied by structural reorganizations associated with EPS production, which are mathematically reflected in changes in surface roughness and vertical height distribution over the effective analyzed area. In this context, parameters such as the root mean square roughness (*S*_q_) and the surface skewness (*S*_sk_) serve as highly sensitive and objective biophysical descriptors of biofilm surface morphology dynamics during growth. By tracking these continuous mathematical shifts, the specific physical milestones of biofilm progression, spanning from early anchoring to late-stage structural reorganization, can be rigorously resolved without relying on destructive multi-staining optical frameworks.

In the present study, *S*_q_ increased progressively from the t0 (control) to t14 (14 days) (Table 4), suggesting a gradual increase in surface heterogeneity and three-dimensional biofilm complexity consistent with the transition toward a mature biofilm stage. The subsequent decrease in *S*_q_ at t21 (21 days) indicates a structural reorganization characteristic of a late-stage biofilm. Complementarily, changes in *S*_sk_ reflected shifts in the asymmetry of the height distribution, evidencing transitions from initial surface colonization toward a more heterogeneous structure and, at later stages, toward a comparatively more homogeneous surface. These results highlight the ability of AFM to capture nanoscale morphological changes that precede and support observations made at larger length scales.

In studies by [55], dry biofilms of *G. sulfurreducens* were analyzed and reported to have an average thickness of approximately 10 μm and a continuous architecture associated with stable biofilm growth. Ref. [56] reported biofilm thickness of up to 34 μm over a period of 6 days using a controlled chemical culture medium. Other studies also employed defined chemical media and achieved biofilms of up to 20 μm, reporting that biofilm thickness can be limited by mass transfer resistance and nutrient diffusion within the matrix [53,54,57,58]. In the present study, biofilms with a maximum thickness of approximately 8 μm were observed, which may be related to the use of whey as a culture medium. This is confirmed by SEM images, which show that whey forms a surface layer; it is hypothesized that this matrix could physically modurate or delay biofilm maturation.

The presence of this whey-derived ‘conditioning surface layer’ suggests that proteinaceous and lipid components from the dairy byproduct may be adsorbing onto the electrode surface alongside the bacteria. This phenomenon is consistent with the restricted biofilm thickness observed throughout the evaluation period, which ranged from 4 to 8 μm (Figure 3). The physical ‘fouling’ effect of the complex whey matrix accounts for this lower thickness (8 μm) relative to synthetic media. Consequently, the structural reorganization observed by AFM at 21 days likely reflects the bacteria’s adaptation to this complex environment, seeking to optimize contact with the graphite surface despite the presence of the whey matrix. Ultimately, this structural limitation represents a reasonable biotechnological trade-off; although standard synthetic media optimize pure spatial development, raw cheese whey excels under a dual-benefit paradigm by validating the viability of alternative carbon source exploration for environmental bioremediation via organic load and chemical oxygen demand (COD) reduction under true circular economy principles [59].

Scanning Electron Microscopy confirms the formation of a *G. sulfurreducens* biofilm on the graphite bar surface, showing relatively continuous coverage and cell accumulations. Previous studies have reported that the porous structure of graphite supports favors *G. sulfurreducens* adhesion and the subsequent establishment of biofilms [55,60]. The images obtained in this study show a more irregular structure, which could be directly associated with the composition of the growth medium (whey). Refs. [16,60] also observed that biofilm density and adherence are highly dependent on the substrate composition. While sample dehydration for electron microscopy may introduce surface artifacts, these qualitative images serve strictly as a correlative macro-scale cross-validation of the continuous topological trends quantified via non-destructive AFM. Thus, the irregular and dense morphology observed in this study reflects how the complex matrix of cheese whey modifies the classic structural development of *G. sulfurreducens* typically seen in defined chemical media.

Energy dispersive spectroscopy (EDS) analysis of the *G. sulfurreducens* biofilm revealed the presence of elements characteristic of both bacterial biomass and the growth medium. The presence of carbon and oxygen may align with the organic compositions of both the bacterial cells and the whey components, alongside the carbonaceous graphite supports [61,62]. Inorganic elements such as sodium (Na), potassium (K), calcium (Ca), magnesium (Mg), chlorine (Cl), and sulfur (S) were also detected and are primarily attributable to the mineral composition of the whey, as previously reported by [52]. In addition, Na and Cl are typical electrolytes, while K and Mg are associated with cellular metabolism. The presence of Ca may be related to the stabilization of the extracellular polymeric matrix (EPS), and sulfur is likely linked to sulfur-containing amino acids in both the bacterial biomass and residual whey proteins [61,63]. On the other hand, the presence of aluminum (Al) is explained by the interaction of the electron beam with the aluminum stub used to mount the sample, a common interference in EDS analysis when the beam penetrates beyond the surface layer [60,64,65,66,67]. Crucially, the definitive tracking of these inorganic elements, particularly potassium (K) which is completely absent in the raw control graphite supports, acts as an essential elemental fingerprint confirming the active integration of the nutrient-rich dairy matrix within the extracellular biofilm boundaries. While other key macro-elements naturally present in whey, such as phosphorus (P), remained below the definitive detection limit of the EDS instrument due to dilution within the organic fractions, their essential metabolic role in driving ATP synthesis and bacterial proliferation is fully supported by literature frameworks [37,38]. While these elements are in biological systems and cellular components, it is important to emphasize that the EDS provides strictly elemental identifications and quantifications. Therefore, there still need studies of evidence of specific extracellular polymeric substances structures or electroactive biomolecules.

## 5. Conclusions

This study shows that pre-treated regional dairy whey can serve as a suitable, nutrient-sufficient standalone substrate for *Geobacter sulfurreducens* biofilm colonization, eliminating the need for expensive synthetic mineral supplements due to its natural phosphorus and potassium content. From an applied perspective, utilizing a standardized regional composite matrix proves that variations in artisanal manufacturing can be biologically mitigated through pooling strategies. Future developments should focus on scaling up this bio-electrochemical system under continuous flow, where optimization of the organic loading rate will be essential to translate these fundamental colonization insights into viable decentralized energy-recovery technologies for the dairy sector.

Whey proved to be a viable substrate for *Geobacter sulfurreducens*, supporting complex diauxic-type growth and the formation of a dense, continuous biofilm on graphite rod electrodes. Despite the organic complexity of the byproduct, the biofilm reached a thickness of 8 μm, demonstrating that stabilized dairy waste can successfully support the development of robust microbial architectures on solid matrices without the need for expensive synthetic media.

The integration of SEM and AFM revealed a nonlinear topographic evolution, where a peak in structural heterogeneity at 14 days was followed by reorganization and stabilization in the late stages at 21 days. While EDS confirmed a rich mineral and organic matrix derived from whey, the observed “conditioning layer” suggests that substrate components can modulate the biofilm-substrate interface, facilitating initial cellular adhesion.

Overall, these morphological and kinetic findings confirm that cheese whey is a highly suitable candidate for biotechnological valorization processes utilizing pure cultures of *G. sulfurreducens*. This study establishes a fundamental structural baseline for future research focused on evaluating and optimizing the performance and long-term stability and ecological resilience of exoelectrogenic biofilms when exposed to dense, actual industrial effluents under sterile conditions.

## Figures and Tables

**Table 1:** One-way ANOVA for macromolecular compositional variability among the five local dairy producers (n = 90).

Variable (%)	Source of Variation	Degrees of Freedom (DF)	F-Value	*p*-Value	Statistical Significance
Fat	Between producers	4	97.379	<0.001	*** (Significant)
	Within error	85			
	Total	89			
Protein	Between producers	4	11.639	<0.001	*** (Significant)
	Within error	85			
	Total	89			
Lactose	Between producers	4	15.473	<0.001	*** (Significant)
	Within error	85			
	Total	89			
Total Solids	Between producers	4	15.361	<0.001	*** (Significant)
	Within error	85			
	Total	89			

Note: *** Significant differences at α = 0.05.

**Table 2:** Physicochemical composition of the standardized cheese whey matrix. Data are expressed as mean ± standard deviation (n = 3).

Parameter	Composite Lot	Reference Literature Values (Regional Context)
Lactose (%)	4.422 ± 0.009	
Total Protein (%)	1.062 ± 0.005	
Total Fat (%)	0.9 ± 0.008	
Total solids (%)	6.54 ± 0.061	
COD (mg/L)		50,000–80,000 [36]
BOD (mg/L)		37,000–60,000 [36]

**Table 3:** Kinetic parameters of *G. sulfurreducens* in whey-based and NBAF media.

	Growth Phase	μ (h^−1^)	g (h)	n
Whey-based	Phase I (0–24 h)	0.02	34.66	0.69
	Phase II (48–72 h)	0.01	69.31	0.34
NBAF (control)	Phase I (0–24 h)	0.001	693.14	0.0002

**Table 4:** Surface roughness (S_q_) and skewness (S_sk_) parameters during *G. sulfurreducens* biofilm development.

Incubation Time (Days)	Root Mean Square Roughness (S_q_ nm)	Surface Skewness (S_sk_)
8	300.74 ± 43.71	1.29 ± 0.52
14	683.08 ± 397.04	2.47 ± 0.71
21	419.73 ± 120.46	1.02 ± 1.28

Note: Values represent the mean ± standard deviation (SD) calculated over an effective area of 68 × 68 μm^2^. S_q_ indicates the vertical deviations of the surface, while S_sk_ describes the asymmetry of the height distribution (positive values indicate a predominance of peaks/clusters).

## Data Availability

The data presented in this study are available on request from the corresponding author.

## Abbreviations

AFM	Atomic Force Microscope
SEM	Scanning electron microscopy
EDS	Energy-Dispersive X-ray Spectroscopy
BOD	Biochemical oxygen demand
COD	Chemical oxygen demand
MFC	Microbial Fuel Cell
FTIR	Fourier Transform Infrared
OD	Optical Density
cps/eV	counts per second per electron volt
keV	kiloelectron volts
Sq	Surface roughness
Ssk	Asymmetry parameter
